# Biometric and gonadosomatic indices and chemical constituents of edible tissues and exoskeletons of *Callinectes amnicola* and their potential for reuse in the circular economy paradigm

**DOI:** 10.1038/s41598-023-35732-1

**Published:** 2023-05-25

**Authors:** Toheeb Lekan Jolaosho, Isa Olalekan Elegbede, Shehu Latunji Akintola, Abayomi Abdul-Azeez Jimoh

**Affiliations:** 1 Department of Fisheries, Lagos State University, Lagos Badagry Expressway, P.M.B. 0001, Ojo, Nigeria; 2 Department of Environmental Planning, Brandenburg University of Technology, Cottbus, Germany; 3 Department of Marine Sciences, University of Lagos, Akoka, Lagos Nigeria

**Keywords:** Biological techniques, Biotechnology, Chemical biology, Structural biology, Ecology, Environmental sciences, Energy science and technology

## Abstract

The study investigates some biological indices and chemical compositions of Callinectes amnicola and their potential for reuse in the context of the circular economy paradigm. The total of 322 mixed-sex C. amnicola collected over a period of six months was examined. The morphometric and meristic characteristics were estimated for biometric assessment. The gonads were obtained from the female crabs for gonadosomatic indices. The shell was obtained using the hand removal technique by detaching it from the crab body. The edible and shell portions were processed separately and subjected to chemical analysis. Our findings showed that females had the highest sex ratio across the six months. The slope values (b) for both sexes exhibited negative allometric growth across all months since the slope values obtained were less than 3 (b < 3). The values obtained for Fulton’s condition factor (K) of crabs in all examined months were greater than 1. The edible portion had the highest moisture level at 62.57 ± 2.16% and varied significantly (*P* < 0.05). The high amount of total ash obtained in the shell sample showed that ash is the main mineral present in crab shells and showed a significant difference (*P* < 0.05). The shell sample had the highest concentrations of Na and CaCO_3_. Based on the findings of this study, it was observed that the shell waste contains some essential and transitional minerals (Ca, CaCO_3_, Na, and Mg) and can be utilized as catalysts in several local and industrial applications, such as pigments, adsorbents, therapeutics, livestock feeds, biomedical industries, liming, fertilization, and so on. Proper valorization of this shell waste should be encouraged rather than discarding it.

## Introduction

Crabs are known to be highly essential in human nutrition because they contain some indispensable fatty acids, particularly omega-3 polyunsaturated fatty acids. They are also a good source of valuable nutrients required for body growth, tissue repair, and development^1^. The blue swimming crab (*Callinectes amnicola*) dominates Nigeria's brackish wetlands and lagoons and is one of the most recognized and prioritized species from the family Portunidae due to its commercial and economic importance^2^. Globally, crabs are primarily valued for their high proteins and macro-minerals such as Ca, Mg, and Na, and their importance in livestock feed industries has been ascertained^3,4^.

The socio-economic and nutritional importance^5^, morphometrics^6^, morphology, abundance, size, and sex distribution^7^, and population characteristics^8^ of diverse crab species from Nigerian freshwater systems have been reported. However, variations in the results and conclusions of numerous studies on Nigerian crabs has observed. The dissimilarities in the biological information of crabs in various scientific studies have been attributed to differences in the biophysical and ecological conditions of the study grounds, sampling gear and designs, and the season at which the study was carried out. It is worth noting that the ecological conditions of freshwater ecosystems are mainly influenced by natural and anthropogenic pressures instigated by humans. Exponential declines with respect to fish catch, diversity, abundance, and composition have been experienced in Nigerian freshwater systems in the last couple of years. Fabre et al.^9^ predicted that in a few years to come, most inland freshwaters in Nigeria will no longer be major sources of aquatic foods due to an exponential decline in total catch. This prediction seems unfavorable to the sustainability of fisheries; hence, more information on fisheries resources along with conservative management measures need to be established.

Length–weight relationship (LWR) is an important index used for the evaluation of fish weight and length distribution^10^ in one or multiple aquatic communities. The metrics "a" and "b" are the most important parameters used when quantifying biological growth and stock assessments of aquatic species^11–14^. The concept of length–weight relationship (LWR) is so essential in fisheries management that it could also be used to establish different ascertainments with respect to the biophysical status of aquatic ecosystems and aquatic organisms. Investigations on fecundity have been useful in racial distinction, progeny survival studies, stock assessment, farmed fish-based hypophysation, and egg incubation^15–17^. The distinct attributes of a fish egg, such as shape, color, and shape, are also important in determining the maturity stage of eggs so that the spawning ability and period of the fish can be ascertained^18^. The gonadosomatic index (GSI) is one of the most recognized metrics in fisheries management and is used in reproduction studies of fish. Application of GSI to determine egg abundance, quality, maturity stage, hydrated ovaries, and reproduction time with reference to an increase in weight was established by Hunter and Macewicz^19^.

In recent years, the term ‘blue economy," which aims to maximize the sustainable utilization of aquatic resources, particularly waste products, has spread throughout the fishing world. This framework has further been promoted as a result of the overreliance on crops and several limitations of cropland materials^20^. Few reports have suggested that the seas and oceans contain ineffable resources with a wide range of potential. Unfortunately, these potentials have yet to be fully realized in the manufacturing industries. Shell waste, on the other hand, has been a major stumbling block for the shellfish processing and/or production industries, marketers, and even consumers^21,22^. Shells in general often have low moisture contents and high compactness and hardness, which makes them difficult to biodegrade and, as a result, resistant to quick spoilage. They tend to stay unmodified in the environment for a longer period of time because of this characteristic^23^. Jung et al.^21^ affirmed that shell waste can be found in almost every part of the world due to inappropriate disposal policies. This, however, results in adverse environmental pollution, which includes unpleasant smells and contamination of the terrestrial and aquatic environments as a result of partial or total decay of this biodegradable material. Since shells are readily available at no or low cost, maximum utilization of shells should be encouraged without ignoring the aftermath while putting in place sustainable management of shell waste in fisheries. Utilization of shells in various value-added strategies would enhance the sustainability of the fisheries, boost the economy, and also contribute to a nation’s food security in the coming years^24^.

Shell waste represents an essential resource in the current "zero waste" movement for a sustainable circular economy, such that this indispensable material could be utilized to achieve economic goals while minimizing environmental pollution. Shells contain high CaCO_3_ and could be partially used as supplementary natural materials in several industrial applications^25^. Shells have been used in several applications^24,26,27^. This resource has been used specifically for soil conditioning^28,29^ and building materials^30,31^. As a result, the processed shell of C. *amnicola* should be utilized in several industrial applications, particularly as a premix for the production of livestock and fish feed. This would further help in the achievement of the zero-waste plans. Furthermore, it will help to strengthen the development of aquaculture from an economic standpoint and assist the fish farmers in alleviating the burden of feed component expenses.

In essence, the idea of reusing food waste to ensure zero waste is one of the major targets of the European Commission’s 2015 Circular Economy Action Plan, which was later upgraded in 2020^22^. Their goals aligned with the context of sustainable development goals such as reducing poverty and hunger, creating long-term jobs through the reuse of food waste, and building a low-carbon economy^32^. For many years, the "long-shared linear economy model", has been utilized to secure unrestrained exploitation of already finite natural resources while mishandling large byproducts that could be valorized^22,33^. The fundamental principle required to ensure byproduct utilization while achieving the sustainable circular economy’s zero waste target is waste valuation. Apparently, the main byproduct of mollusks is their exoskeletons, but with proper management, they can find a legitimate way into the paradigm of "zero waste" for the circular economy through a variety of applications regardless of the distribution chains^22^.

Numerous valorization strategies for shell waste have been reported in several studies^24,27–31,34–36^^.^ Nonetheless, this byproduct has yet to reach its full potential in the context of a sustainable circular economy. Summa et al.^22^ maintained that several circularity papers have been published in recent years with a focus on effective shell waste reuse in several industries; this denotes a growing interest in zero waste frameworks. The techno-economic and sensitivity return assessment of Olivier et al.^37^showed that the inclusion of shell waste could help save up to $7.0 billion per year, provided it is utilized to its full potential.

Calcareous shells are produced in large quantities by the shellfish processing industry^24,26,27^. Improper disposal procedures for large quantities of shell waste have been a major constraint for these industries^38^. Shell waste dumped in inland waters causes environmental pollution such as changes in water quality, destruction of natural landscapes, and instability of the environment’s biophysical and ecological status^27,39^. Shellfish waste generated by shellfish aquaculture-based firms or capture fisheries should be recycled or valorized to reduce environmental pollution while meeting the "zero waste" goal of the blue circular bio-economy. Due to insufficient scientific knowledge, the global perception of most people is that shells detached from crabs such as bivalve and shrimp are not useful; however, valorizing these non-essential materials could turn them into high-value-added products^26^.

Studies on the valorization potential of shells such as oysters and bivalves in several industrial applications have been documented. However, no scientific study has been conducted on the valorization and industrial potentials of C. *amnicola* shell and how it could be incorporated into several applications. This paper becomes imperative and thus highlights the biometric information of crabs required for sustainable fisheries management and also estimates the importance of crab shell waste and how it could be applied in several applications in the context of the sustainable circular economy paradigm in order to lessen byproducts or waste generated from fisheries and to ensure a more effective production structure. The study, however, looks into morphometrics and meristics, length–weight relationships, condition factors, gonadosomatic indexes, and sex ratios, as well as the chemical compositions of *Callinectes amnicola* and their potential for reuse in the context of the circular economy paradigm.

## Materials and methods

### Sampling location

**Figure 1:**
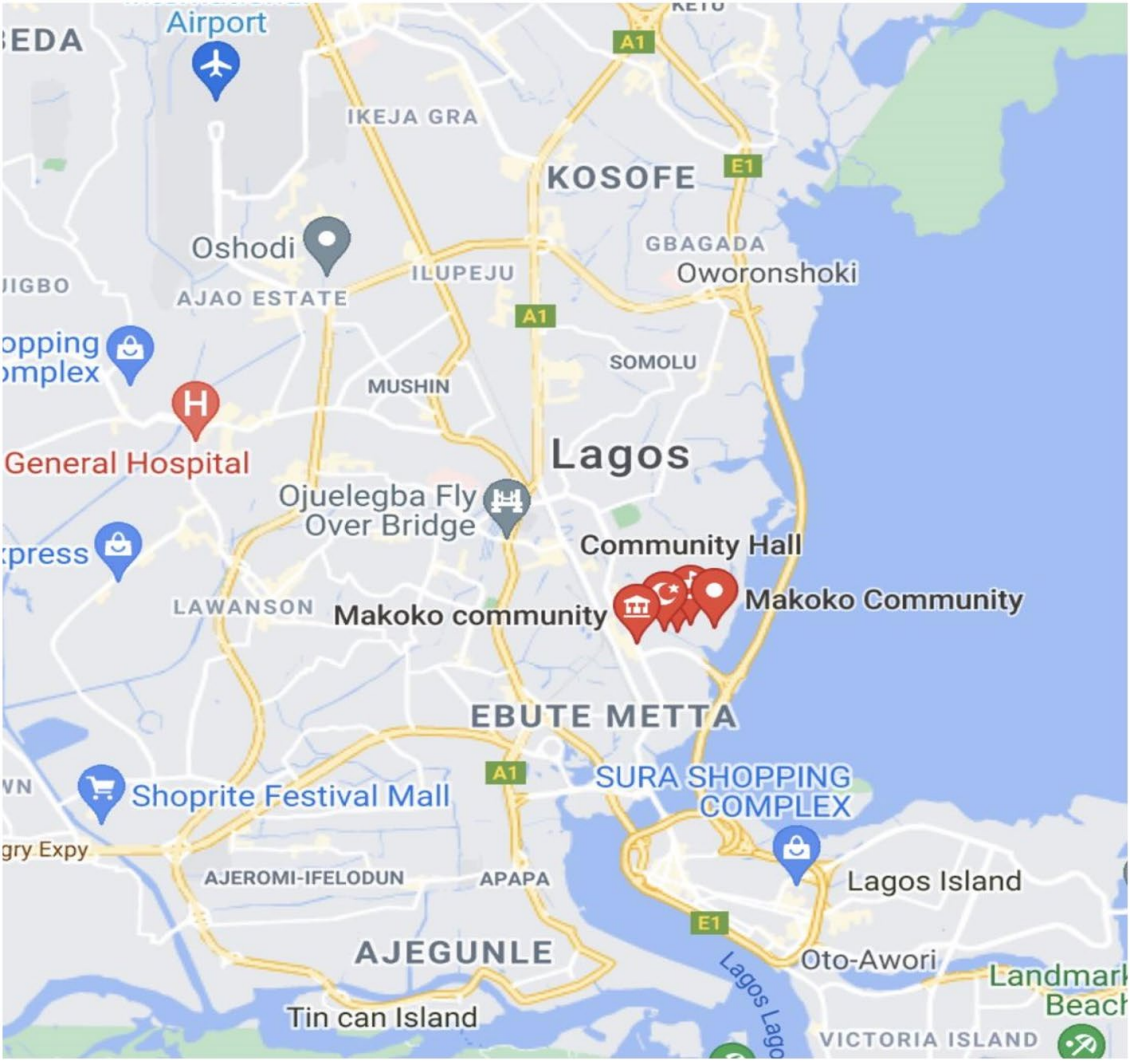
Map of the Makoko fishing community indicating the sampling site (Source: Google).

The study was conducted at the Makoko fish landing site in the mangrove swamp bordering the Abule-Eledu Creek, which forms part of the many sluggish tidal creeks that drain into the Lagos Lagoon at Yaba Local Government Area of Lagos State, Nigeria (Fig. 1). Makoko in Lagos State is located within the coordinates of Latitude 6.29ºN and Longitude 3.23º E of the Equator, which is about 1.53 miles away from the University of Lagos. The river serves as one of Lagos State’s major fishing grounds. The market (Makoko market) is located beside the river, which enables the populace of Yaba and its environs to obtain fresh fish readily on a daily basis.

### Collection of experimental samples

The crab samples used in this study were purchased directly from the artisanal fishermen, who operate mainly with non-mechanized gears such as basket traps, wire traps, and circulation lift nets. A total of 322 mixed crab samples were examined. The sample collection from the sampling site was performed once a month for a period of six months, although not for six consecutive months (January 2021 to November 2021). The reason for the inconsistency in sampling months was due to the non-availability of the experimental species in fishermen’s catch in some months. The sampling was done between 7:00 a.m. and 10:00 a.m. on each occasion of collection. The crabs collected were transported to the laboratory of the Department of Fisheries, Lagos State University, in a plastic ice chest box to prevent spoilage during transportation.

### Morphological identification and measurement

**Figure 2:**
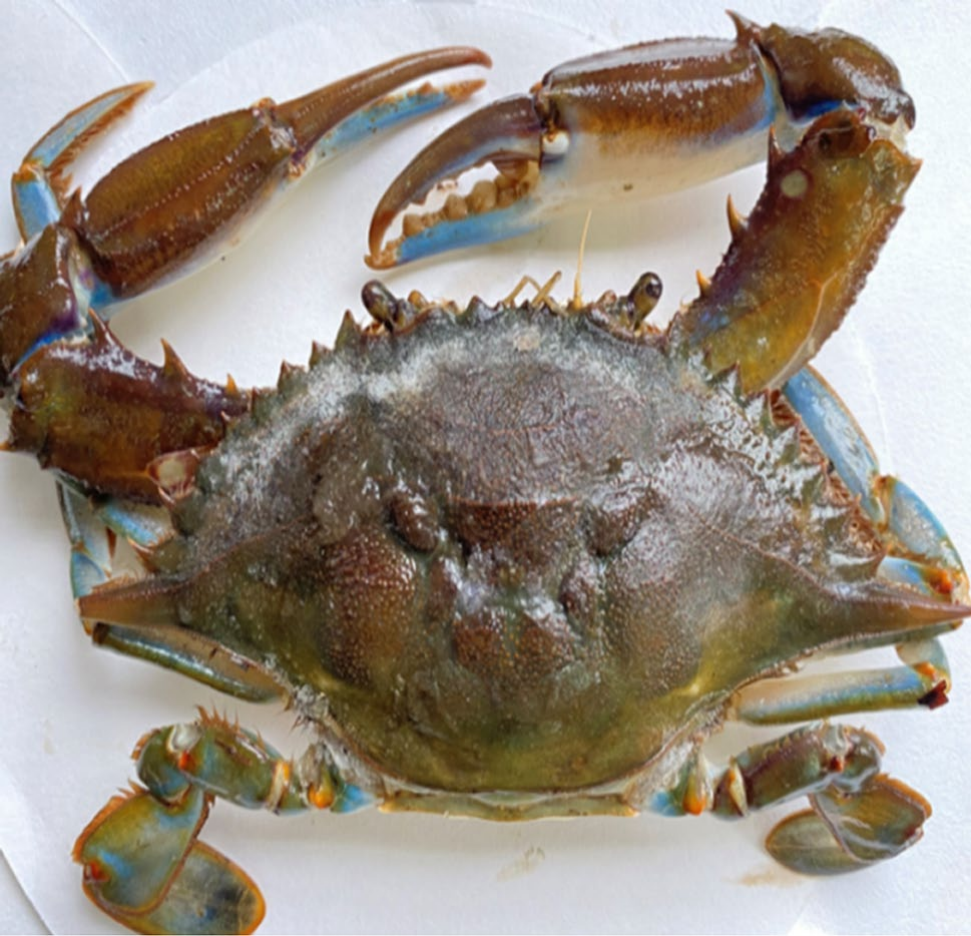
Dorsal view of *Callinectes amnicola.*

**Figure 3:**
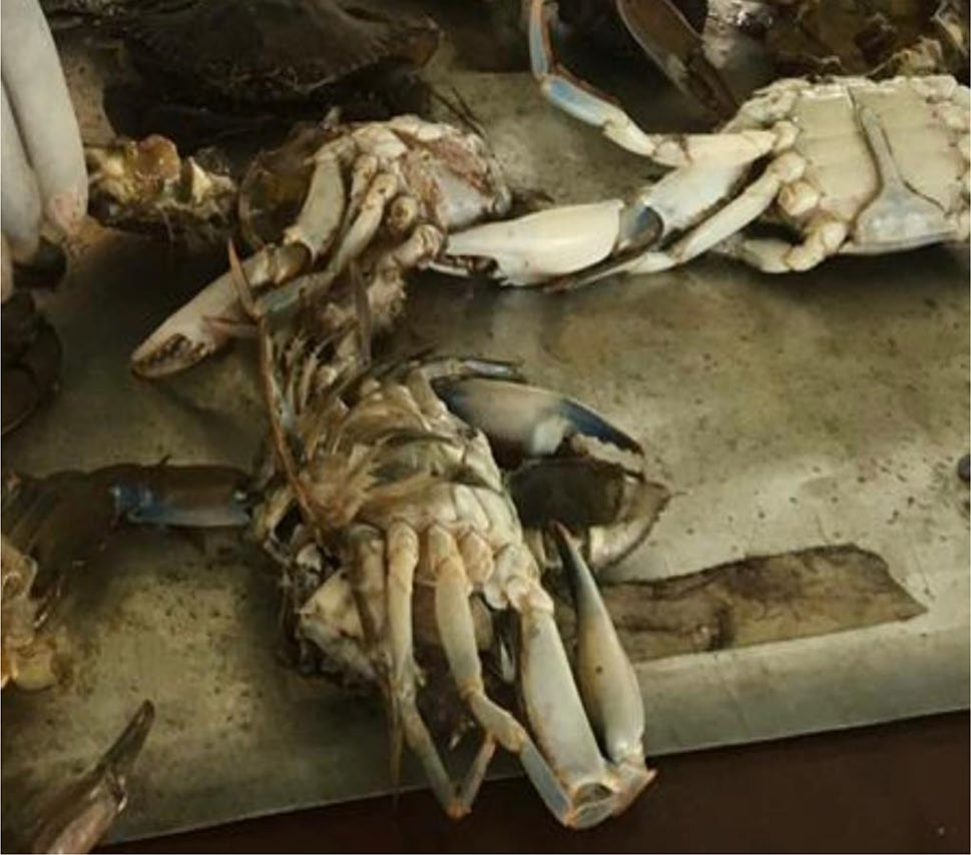
Ventral view of *Callinectes amnicola.*

The illustrated method of Kwei^40^ was adopted in order to ensure appropriate species identification such as taxonomic classification. The experimental species to be assessed were sorted based on sex differences (male and female). For morphological classification, visible external features such as the T-shaped abdomen in males and the triangular or rounded aprons in females were used to distinguish the species^40^. In Fig. 2, the dorsal view of *C*. *amnicola is presented,* while the ventral view is displayed in Fig. 3. Prior to weighing, the crabs were dried to ensure total removal of excess water and obtain an accurate weight of the specimen. The morphometric features such as carapace length (cm) and total weight (g) were estimated such that the instrument (a sliding vernier caliper) used for the carapace length measurement was calibrated to the nearest 0.1 cm, while an electric weighing balance (model PM400) calibrated to the nearest 0.01 g was used to quantify the body weight.

### Length–weight determination

The Length–weight relationship (LWR) was evaluated from the regression equation^10^:

1 $${\text{W}} =\,_{{\text{a}}}{\text{L}}^{{\text{b}}}$$

where W denotes the total body weight (g), L represents the total length (cm), ‘’a’’ means intercept, b represents the growth coefficient.

To obtain Linearity of data, a transformed logarithmic equation (least square linear regression) illustrated below was adopted

2 $${\text{Log}}\,{\text{W}} = {\text{Log}}\,{\text{a}} + {\text{b}}\,{\text{Log}}\,{\text{L}}$$

where Log denotes the natural log; The "b" value was used to determine the growth pattern and was interpreted as follows: b = 3 means isometric growth; b > 3 denotes positive allometric growth; and b 3 represents negative allometric growth.

### Fulton’s condition factor

The condition factor (K) was estimated through the correlation of the carapace length and body weight of the crabs measured with respect to the equation below^3,11^:

3 $${\text{K}} = 100\,{\text{W}}/{\text{L}}$$

where K is the condition factor (cf), W is the total body weight (BW), L is the carapace length (CL), 3 is a constant.

### Sex ratio

Based on the number of fish samples, the equation below was used to compute the sex ratio^41,42^.

4 $$\chi 2 = \sum \left( {{\varvec{O}}_{{\varvec{i}}} {-}{\varvec{E}}_{{\varvec{i}}} } \right)^{2} /{\varvec{E}}_{{\varvec{i}}}$$

where ***O***_***i***_ is the observed value, ***E***_***i***_ is the expected value

### Morphometric and meristic parameters/codes

The morphometric and meristic parameters and the definition of the characters measured were presented in the Table 1.

**Table 1:** Morphometric and meristic character codes.

S/N	Morphometric & meristic variables	Codes	Definition/Meaning
1	CARAPACE WIDTH	CW	The distance between lateral spines of the carapace
2	CARAPACE LENGTH	CL	Straight line measurement from the rear of the eye socket parallel to the centre line to the posterior edge of the carapace
3	ROSTRUM LENGTH	RL	Distance from the tip of the rostrum to the postorbital margin of the carapace
4	ROSTRUM WIDTH	RW	Distance from one end of the side of the rostrum to another
5	BODY WEIGHT	BW	Entire weight of crab
6	RIGHT DACTYL CHELA TEETH UPPER	RDCTU	Total number of Teeth at the upper Right-hand side
7	RIGHT DACTYL CHELA TEETH LOWER	RDCTL	Total number of Teeth at the lower Right-hand side
8	LEFT DACTYL CHELA TEETH UPPER	LCDCTU	Total number of Teeth at the upper left-hand side
9	LEFT DACTYL CHELA TEETH LOWER	LDCTL	Total number of Teeth at the lower left-hand side

Source: Gunawickrama^43^, Howland et al.^44^.

### Gonadosomatic index

The female crabs were dissected by opening the abdominal ventral cavity. The shape of the abdomen was used in identifying the female crabs. The gonadosomatic index (GSI) was calculated only for the female crabs using the formula of Howaida et al.^45^ below.

5 $${\text{GSI}} = \frac{{{\text{Weight}}\,{\text{of}}\,{\text{Gonads}}}}{{{\text{Body}}\,{\text{Weight}}\,{\text{of}}\,{\text{fish}}}} \times 100$$

## Laboratory procedures

### Retrieval process of the edible part and shell waste for analyses

The shell was detached from the crab body using the hand method. The edible and shell portions were processed separately, such that localized pestles and mortars were utilized to grind the edible and shell portions until they turned into powdery forms. The finely ground samples were dried afterwards in a mechanical oven for about 3 h at 105 °C. The finely ground dried chitosan powder of the samples was rigorously mixed with potassium bromide (KBr) and compressed using a pellet gun to develop a few similar discs of 0.5 mm thickness^46^. The prepared chitosan, developed in the form of homogenous discs, was characterized with a Fourier Transformed Infrared (FT-IR) spectroscopy instrument (Bruker Alpha-T). The instrument** ‘**FT-IR Spectroscopy’ at a density of 4000–400 cm1 and acid–base titration techniques were adopted for the quantification of the degree of deacetylation (DD). The characterized chitosan samples were placed in a sealed desiccant container for 12 h prior to scanning^47^. The approach of Kalutharage et al.^47^ was used to estimate the DD of the chitosan samples. The processed samples were analyzed for chemical compositions.

### Procurement of reagents

The reagents used for the experiment include:

The reagents NaOH and HCL were utilized for the purification of samples and the prevention of sample spoilage, respectively. H_2_SO_4_ was adopted for both oxidation and sample dehydration. Additionally, NaClO was used to disinfect the surface or object prior to analysis, while CH_2_Cl_2_ was used as a solvent during analysis.

### Proximate analysis

The procedures recommended by the Association of Official Analytical Chemists (AOAC,^48–52^) were used.

### Determination of moisture content

The moisture content was evaluated by drying empty crucibles in a standard oven at 105 ± 5 °C for 30 min to eliminate the moisture present. The empty, dried crucibles were weighed and recorded as W_0._ 1.00 g of the grinded chitosan was weighed and transferred into the empty crucible, where it dried in the standard oven at 105 ± 5 °C for 4 h. The heated crucible containing the powdered chitosan was allowed to cool for 10 min, weighed, and tagged as W_1_. The crucibles containing the samples were transferred into an enclosed container that contains desiccant and allowed to cool at room temperature for about 30 min, after which they were weighed and recorded as W_2_.

The moisture content was computed using the equation^51^:

6 $$\% {\text{Moisture}}\,{\text{content}} = \frac{{\left( {W_{0} + W_{1} } \right) - \left( {W_{0} + W_{2} } \right)}}{{W_{2} }} \times 100$$

where W_0_ = weight of empty dried crucibles, W_1_ = initial weight of sample in crucibles, W_2_ = final weight of samples in the crucibles.

### Determination of protein

The Kjeldahl block digestion and steam distillation methods were adopted to determine the protein content. 0.1 g of methyl red and bromocresol green indicators were dissolved in 100 ml of 95% methanol to prepare the solution. A boric acid solution at 4% was prepared, and 400 g of the chitosan powder was dissolved in 5 L of extremely hot distilled water and allowed to cool. Bromocresol and methyl red solutions, measured at the rates of 100 and 70 ml, respectively, were included in the solution, and 10 L of deionized water was added and thoroughly mixed. 1 g of solution was measured into a 250-ml digestion tube; 2 Kjeltabs Cu 3.5 and 12 ml of concentrated H_2_SO_4_ were also included. The exhaust system was fastened to the digestion tubes, which were placed in a rack, and the water aspirator was configured to full effect. The prepared sample underwent digestion for 1 h at 420 °C. The rack on which the digested tube containing the sample was placed was withdrawn and allowed to cool for 15 min. After cooling, 80 ml of deionized water was added to the solution in the tube, which was then transferred into the distillation unit. A 25-ml receiver solution was added to the conical flask and inserted into the distillation unit. A pathway underneath was created so as to ensure that a distillate channel is submerged in the receiver solution. 50 ml of 40% NaOH was added to the solution and distilled for 4 min. The distillate was then titrated with systematized HCL (usually 0.1 or 0.2 N) until the blue-grey end point was obtained.

7 $$\% Crude\;Protein = \frac{{\left( {T - B} \right) \times N \times 14.007 \times F}}{{W_{1} \,({\text{mg}})}} \times 100\quad \left( {{\text{EN}}\,{\text{ISO}\,20483{:}2006}} \right)$$

$$gN/L = \frac{{\left( {T - B} \right) \times N \times 14.007}}{{Volume_{sample} \left( {{\text{ml}}} \right)}}$$

where W_1_ = sample weight (mg), T = Titration volume of sample (ml), B = Titration volume of blank (ml), N = Normality of acid to 4 decimal places, F = conversion factor for nitrogen to protein = 6.25 for food & feeds, gN/L = Gram Nitrogen per litre

### Determination of crude fat

The Soxhlet extraction technique was adopted to estimate the crude fat content present in samples. A finely ground chitosan sample of 5.00 g was measured into a metal ferrule, while cotton wool was used as a shielding material so as to ensure the chitosan sample did not pour out during the extraction processes. The round-bottom flask was thoroughly dried at 60 °C and weighed. 80 ml of hexane was poured into the flask, and the small metal ferrule in which the chitosan sample was contained was placed into the extractor. The heating mantle was activated, and water was set to run through the condenser to ensure cooling. The extraction was allowed to undergo its reverse flow for exactly 2 h, after which it was stopped. The quantity of crude fat oil (in percentage) present in the sample was obtained by deducting the initial weight from the final weight of the flask^51^.

8 $$\% fat = \frac{weight\;of\;flask\;after\;extraction\;\& \;drying\; - \;weight\;of\;empty\;flask}{{Sample\;weight}} \times 100$$

### Determination of crude fiber using Fibertec

The fiber content was evaluated by allowing the pre-dried sample to be sintered. Crucible through a Fibertec hot/hydrolysis unit and a Fibertec cold extraction unit were adopted. The samples were calcined for 3 h at 525 ± 15º C and allowed to cool prior to weighing^50^.

9 $$\% \;Crude\;Fiber = \frac{{W_{2} - \left( {W_{3} + C} \right)}}{{W_{1} }} \times 100$$

where W_1_ = Sample weight (g), W_2_ = Crucible + residue weight after drying (g), W_3_ = Crucible + residue weight after Ashing (g), C = Blank.

### Determination of crude ash

The crucible was dried in a standardized oven at 130 ± 15ºC for 30 min to obtain a moisture-free crucible. The dried crucible was allowed to cool in desiccators for 15 min, weighed, and noted as W_0_. The finely blended powdery sample achieved with the Cyclotec milling machine was weighed at a rate of 1.00 g with the aid of an analytical balance into the dried crucible and calcined in the furnace at 500 ± 15ºC for 6 h. The sample was weighed and recorded as W_1_. The ash sample was allowed to undergo cooling for 30 min. The heated crucible was transferred into the desiccators with the aid of a scissor-like metal tool known as the crucible tong and allowed to cool for 45 min. The weight of the crucible containing the residue after ashing was then taken as the final weight and recorded as W_2_. The percentage of ash was calculated as follows^49^:

10 $$Ash\;content = \frac{{\left( {W_{2} - W_{0} } \right)}}{{W_{1} }} \times 100$$

### Nitrogen free extract (N.F.E)

All the proximate parameters that are not evaluated are categorized as nitrogen-free extract (NFE). They include digestible carbohydrates, vitamins, and other non-nitrogenous, soluble organic compounds. Since the results are obtained by deducting the percentage obtained for each nutrient from 100.

11 $${\text{N.F.E}}\,\left( \% \right) = 100{-}\left( {{\text{A}} + {\text{B}} + {\text{C}} + {\text{D}} + {\text{E}}} \right)$$

where A = moisture content (%), B = crude protein content (%), C = crude fat content (%), D = crude fibre content (%), E = ash content (%)^48^.

### Determination of mineral components

The atomic absorption spectrophotometer technique was adopted to estimate the mineral contents. 2 g of a powdery dried chitosan sample was measured into a crucible and calcined at 550 oC for 3 h. The crucible was allowed to undergo cooling, after which the ash product was solubilized with 100 ml of 3NHCL. The solution was transferred into a plastic container for AAS readings^52^. For each mineral parameter to be determined, a corresponding lamp was allocated, and a specific wavelength was adopted. The atomic absorption spectrometer (AAS) siphoning tube was fastened into the digested sample, after which the standard wavelength for each mineral to be estimated was processed. The amount of each mineral in the prepared soluble sample was shown on the screen of the AAS digital machine.

### Statistical data for analyses

Microsoft Excel version 15.0 on Windows was utilized to compute the length–weight relationship, morphometric, meristic, condition factor, and GSI data. The mean and standard deviation of chemical contents in samples were also evaluated using Microsoft Excel. SPSS (IBM, 2015) was used to evaluate the regression coefficient. The significant difference in mean and disparity between species was determined using an independent T-test established at 5% (P 0.05).

## Results

The monthly data of the average, minimum, and maximum morphometric characters of *Callinectes amnicola* are presented in Table 2. The highest mean body weight of crabs was obtained in the month of July (142.41 ± 26.75 g). The maximum individual body weight (188.90 g) was obtained in the crabs sampled in the month of January. For carapace length, the month of January, with a value of 2.52–14.12 cm, represented the widest crab range in a cohort. Similarly, the highest carapace length with a value of 0.84 ± 3.53 cm was obtained in the month of January. The highest Gonad Weight (GW) with the value of 24.06 ± 5.91 g, was recorded in the month of July.

**Table 2:** Distribution of average, minimum and maximum morphometric characters of *Callinectes amnicola.*

Month		BW (g)	CL (cm)	CW (g)	RL (cm)	RW (g)	GW (cm)
January	Mean ± Sd Min–Max	104.50 ± 47.08 [5.35–188.90]	9.84 ± 3.53 [2.52–14.12]	7.47 ± 3.69 [2.10–16.93]	1.95 ± 0.60 [0.50–3.10]	2.44 ± 3.13 [0.63–2.92]	18.54 ± 7.96 [0.75–31.84]
March	Mean ± Sd Min–Max	97.49 ± 33.54 [25.52–152.10]	9.43 ± 2.93 [4.85–15.4]	5.16 ± 1.58 [2.52–8.63]	1.9 ± 0.60 [0.94–3.35]	1.79 ± 0.59 [1.00–3.25]	21.66 ± 23.44 [9.22–172.05]
May	Mean ± Sd Min–Max	98.74 ± 39.21 [32.70–170.81]	9.55 ± 3.13 [4.90–15.43]	5.13 ± 1.64 [2.58–8.33]	1.92 ± 0.59 [1.00–2.93]	1.86 ± 0.62 [0.85- 3.00]	17.71 ± 6.65 [6.73–29.34]
July	Mean ± Sd Min–Max	142.41 ± 26.75 [84.25–110.11]	6.11 ± 1.54 [3.00–8.95]	11.73 ± 3.07 [5.23–16.85]	2.54 ± 0.61 [1.26–3.98]	2.46 ± 0.58 [1.00–3.65]	24.06 ± 5.91 [12.25–34.55]
September	Mean ± Sd Min–Max	71.54 ± 32.89 [18.24–163.20]	6.82 ± 10.51 [1.35–7.22]	10.67 ± 4.17 [4.30–18.35]	1.99 ± 0.78 [1.00–3.65]	1.92 ± 0.74 [1.00–3.45]	9.62 ± 4.23 [4.05–20.47]
November	Mean ± Sd Min–Max	76.00 ± 35.2 [23.07–177.94]	5.75 ± 2.00 [2.26–9.15]	11.35 ± 4.00 [4.60–18.38]	2.2 ± 1.0 [1.00–6.95]	2.1 ± 1.0 [1.00–3.85]	10.6 ± 4.8 [4.00–27.25]

Illustrated in Table 3 is the monthly distribution of the average, minimum, and maximum meristic counts of *Callinectes amnicola* from the Makoko axis of Lagos Lagoon. Four (4) meristic characters, as defined in Table 1, were evaluated. The highest mean (RDCTU) of 6.50 ± 1.25 was obtained in March. The maximum (9.50) and minimum (2.00) RDCTUs were obtained in January. The maximum (10.05) and minimum (2.00) values for RDCTL were also obtained in January. Similarly, the highest LDCTU value was recorded in July. The minimum and maximum LDCTL values were observed in the months of January and July, respectively.

**Table 3:** Distribution of average, minimum and maximum meristic count of *Callinectes amnicola.*

Parameters	RDCT (U)	RDCT (L)	LDCT(U)	LDCT(L)
January	4.90 ± 1.40 [2.00–9.50]	4.61 ± 1.70 [2.00–10.05]	4.92 ± 1.53 [3.05–10.00]	4.65 ± 1.63 [2.00–9.00]
March	6.50 ± 1.25 [4.00–9.15]	6.75 ± 1.50 [2.05–8.55]	6.22 ± 1.13 [3.15–8.00]	6.45 ± 1.30 [2.05–8.00]
May	5.45 ± 1.45 [2.10–8.05]	4.85 ± 1.51 [2.65–8.05]	5.12 ± 1.25 [3.12–8.45]	5.00 ± 1.45 [2.32–8.54]
July	6.41 ± 1.25 [4.00–9.12]	6.75 ± 1.22 [4.00–9.00]	6.42 ± 1.15 [4.55–9.05]	6.71 ± 0.90 [5.00–9.15]
September	6.20 ± 1.35 [4.00–9.05]	5.80 ± 1.55 [3.50–8.55]	5.70 ± 1.30 [3.05–8.01]	5.62 ± 1.52 [2.10–9.00]
November	5.70 ± 1.41 [3.00–9.05]	5.71 ± 1.80 [2.05–8.00]	5.40 ± 1.45 [3.00–8.00]	5.60 ± 1.80 [2.10–9.00]

Table 4 depicts the monthly sex distribution, sex ratio, and Chi-Square analysis of *C. amnicola* from the Makoko axis of Lagos Lagoon. The highest sample abundance was obtained in the first and fourth sampled months, with 61 crabs apiece. The female sex had the highest number of samples, coinciding with the highest sex ratio across the six months. The calculated values (X^2^ Cal) obtained for samples across all months were significantly higher than the tabulated values (X^2^ Tab) (3.841). These results indicated that the female crab samples were abundant over the male at (*P* < 0.05).

**Table 4:** Sex Distribution, Ratio and Chi-Square analysis of *Callinectes amnicola* in the monthly samples from Makoko axis of Lagos Lagoon.

Month	No of crab [combined sexes]	Expected [E]	No of M [Observed]	No of F [Observed]	Sex ratio M:F	[O-E]^2^ E	X^2^ CAL	X^2^ TAB [*P* < 0.05]
January	61	30.5	8	53	1.0:6.6	16.60	33.2	3.841
March	56	28	12	44	1.0:3.7	9.14	18.28	3.841
May	45	22.5	5	40	1.0:8.0	13.61	27.22	3.841
July	61	30.5	15	46	1.0:3.1	7.88	15.76	3.841
September	43	21.5	5	38	1.0:7.6	12.66	25.32	3.841
November	56	28	4	52	1.0:13.0	20.57	41.14	3.841

The result of the monthly growth pattern with respect to length–weight relationship using linear regression, condition factor, correlation coefficient, morphemetric data, and related statistics for male, female, and combined sexes of *Callinectes amnicola* is presented in Table 5. As shown in Table 5, the slope values (b) for both sexes exhibited negative allometric growth across all months since the slope values obtained were less than 3 (b < 3). As seen in Table 5, the female exhibited the highest slope (b) value in the months of January, May, September, and November. The values obtained for Fulton’s condition factor in all the months were greater than 1, indicating that the crabs are in good condition in their environment, The highest correlation coefficient ‘r’ between log length and log weight was found to be 0.955 in males in March.

**Table 5:** Length weight relationship using linear regression, Condition factor, Correlation coefficient and morphemetric data of *Calinectes amnicola* from Makoko axis of Lagos Lagoon.

	n	a	b	k	r	r^2^
January						
Female	52	1.049	1.077	16.47	0.727	0.739
Male	8	1.492	1.373	42.3	0.518	0.539
Combined sex	60	0.755	1.20	19.97	0.330	0.349
March						
Female	43	1.288	0.695	15.9	0.300	0.322
Male	12	1.053	0.986	17.40	0.955	0.972
Combined sex	55	1.225	0.771	16.22	0.413	0.429
May						
Female	39	0.757	1.247	14.4	0.817	0.841
Male	5	0.947	1.105	19.0	0.742	0.760
Combined sex	44	0.786	1.22	14.94	0.805	0.827
July						
Female	45	1.901	0.257	82.85	0.378	0.399
Male	14	1.842	0.322	81.23	0.412	0.430
Combined sex	59	1.883	0.277	80.97	0.385	0.407
September						
Female	37	1.230	0.850	98.79	0.584	0.606
Male	5	1.493	0.316	37.60	0.276	0.300
Combined sex	42	1.176	0.917	92.08	0.590	0.614
November						
Female	51	1.049	1.077	60.55	0.717	0.739
Male	4	0.934	1.401	110.51	0.725	0.744
Combined sex	55	1.069	1.057	64.2	0.760	0.782

The percentage gonad weight in relation to total weight, also termed the gonadosomatic index (GSI), was calculated for each month as illustrated in Table 6. During the first month, the value of GSI showed a highest level of 20.82 g, and in the next two months, March and May, the values obtained exponentially decreased to 18.66 g and 18.30 g. From the month of July to November, the values of GSI showed alternate increases, as seen in Table 6. The month of September had a value of 13.89 g, representing the lowest G.S.I. value recorded.

**Table 6:** Monthly variations in Gonado-somatic index (GSI) of *Callinectes amnicola* from Makoko axis of Lagos Lagoon.

Months	Number of females	GSI (Female)
January	52	20.82 ± 2.56
March	43	18.66 ± 1.78
May	39	18.30 ± 1.95
July	45	18.51 ± 2.12
September	37	13.89 ± 1.09
November	51	14.00 ± 1.15

**Figure 4:**
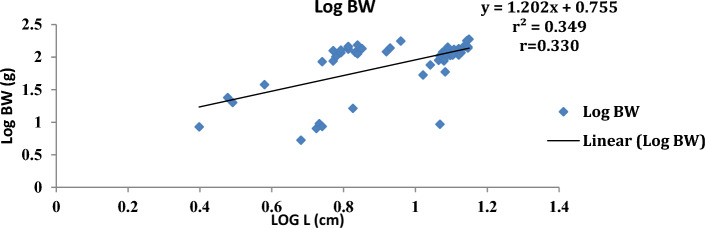
Logistic length weight relationship of *Callinectes amnicola* (Pooled sex) for January, 2021.

**Figure 5:**
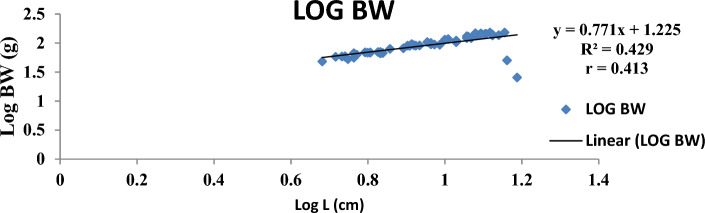
Logistic length weight relationship of *Callinectes amnicola* (Pooled sex) for March, 2021.

**Figure 6:**
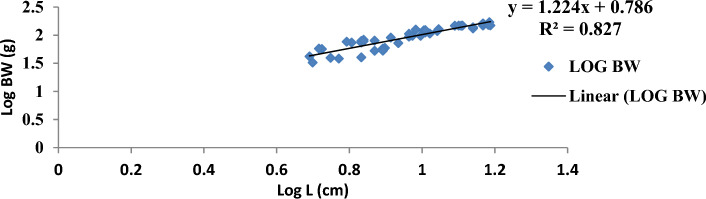
Logistic length weight relationship of *Callinectes amnicola* (Pooled sex) for May, 2021.

**Figure 7:**
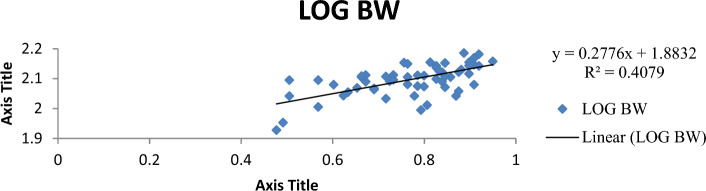
Logistic length weight relationship of *Pseudotolithus typus* (Pooled sex) for July, 2021.

**Figure 8:**
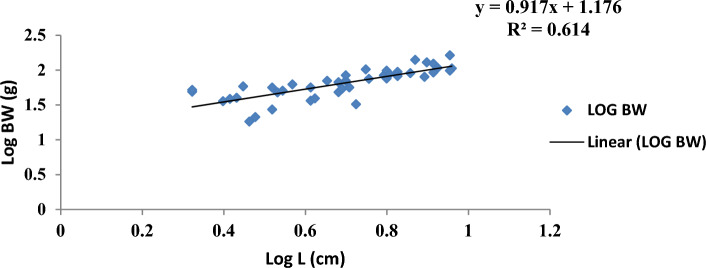
Logistic length weight relationship of *Callinectes amnicola* (Pooled sex) for September, 2021.

**Figure 9:**
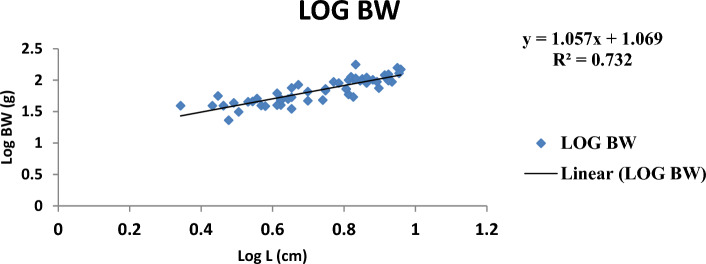
Logistic length–weight relationship of *Callinectes amnicola* (Pooled sex) for November, 2021.

Figures 4, 5, 6, 7, 8 and 9 show the logistic illustration of the length–weight relationship of *Callinectes amnicola* (Pooled sex) for all the months sampled. Figure 4 showed that a negative growth pattern (b < 1.202) was obtained in the month of January. The graph also showed a low positive correlation (0.330) between length and weight. Figure 5 showed that a negative growth pattern (b < 0.771) was obtained in the month of March. The graph also showed a low positive correlation (0.413) between length and weight. A negative growth pattern and strong positive correlation (0.805) were obtained for May, as shown in Fig. 6. Figure 7 showed that a negative growth pattern (b < 0.277) was obtained in the month of July. The graph also showed a low correlation (0.385) between length and weight. Figure 6 also showed a negative growth pattern for the month of September. The graph also showed a low positive correlation (0.590) between length and weight. Lastly, Fig. 9 showed both a negative growth pattern and a positive correlation between length and weight for the month of November.

**Figure 10:**
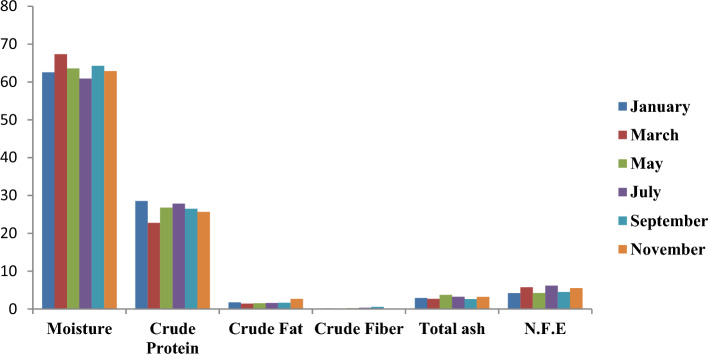
Monthly variation of proximate composition of edible part of *C*. *amnicola.*

Figure 10 illustrates the graphical representation of the monthly distribution of proximate composition in edible parts of *C*. *amnicola*. As presented in Fig. 10, the highest moisture content in the edible part of crab was recorded in March with a value of 67.32%. The highest and lowest crude protein contents in the edible portion were 28.53% and 22.76%, respectively, in January and March. The highest total ash content in the sample was obtained in the month of May.

**Figure 11:**
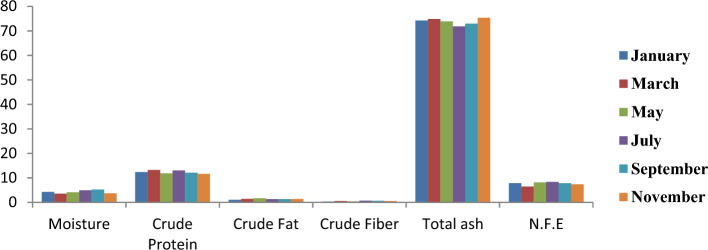
Monthly variation of proximate composition of shell of *C*. *amnicola.*

Figure 11 depicts the monthly variations of proximate composition in the shells of *C*. *amnicola*. The highest moisture content in shell was recorded in September, with a value of 5.22%. The highest and lowest crude protein contents were 13.22% and 11.62%, as seen in shell samples obtained in the months of March and November, respectively. The most abundant crude fiber content was recorded in samples obtained in July at 0.67. A significant amount of total ash contents was obtained across all the sampled months, which showed that total ash is the major constituent of crab shells, such that it ranged from 71.83% to 75.36%.

**Figure 12:**
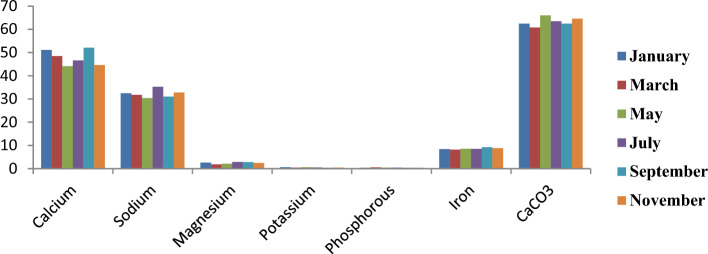
Monthly variation of mineral composition of edible part of *C*. *amnicola.*

Figure 12 shows the monthly variation of mineral contents in the edible portion of *C*. *amnicola*. The figure showed that Ca, Na, and CaCO_3_ are the major minerals present in the edible parts of *C*. *amnicola*. The highest magnesium content (2.59 mg/kg) was recorded in the first month. The samples obtained in the months of January and March had the highest K and P, with 0.58 mg/kg and 0.53 mg/kg, respectively.

**Figure 13:**
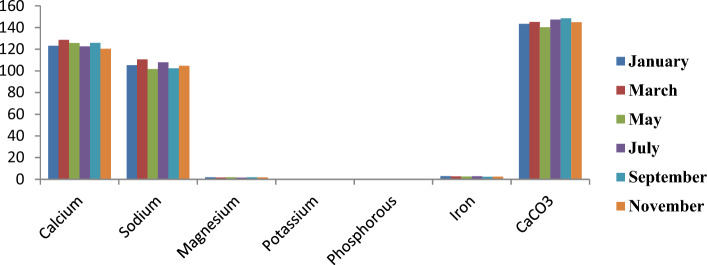
Monthly variation of mineral composition of shell part of *C*. *amnicola.*

Figure 13 illustrates the graphical representation of the monthly distribution of mineral contents in the shell of *C*. *amnicola*. The dominant mineral contents obtained in the shell of this study are Ca, Na, and CaCO3, which are similar to the major minerals present in the edible part of *C*. *amnicola*. The highest Na content was recorded in a shell sample obtained in March with 110.54 mg/kg, while the highest CaCO3 content was recorded in a sample obtained in September with 148.39 mg/kg. Low K and P contents were recorded in all sampled months, ranging from 0.21 to 0.28 mg/kg and 0.08 to 0.15 mg/kg, respectively.

Table 7 depicts the proximate compositions of the edible tissue and shell parts of *C*. *amnicola*, indicating that some of the results were significantly different (*P* < 0.05) from each other. The edible portion had the highest moisture level at 62.57 ± 2.16% and varied significantly (*P* < 0.05). High crude protein was found in the edible tissue of *C*. *amnicola* with 26.34 ± 2.02%, while the lowest concentration was recorded in the shell with 12.35 ± 0.64%, although no significant difference (*P* > 0.05) exists. Relatively similar fat contents were obtained in both edible tissue (1.76 ± 0.46%) and shell (1.35 ± 0.20%). In contrast, the shell sample had the highest crude fiber content with 0.51 ± 0.14% and showed a significant difference (*P* < 0.05). The high amount of total ash obtained in the shell sample (73.85%) showed that ash is the main mineral present in the shell of the crab evaluated in this study, and significant variation (*P* < 0.05) exists.

**Table 7:** Mean concentration of proximate composition of the edible and shell part of *C*. *amnicola.*

Parameters	Edible portion	Shell
Moisture	62.57 ± 2.16^a^	4.29 ± 0.66^b^
Crude Protein	26.34 ± 2.02^a^	12.35 ± 0.64^a^
Crude Fat	1.76 ± 0.46^a^	1.35 ± 0.20^a^
Crude Fiber	0.23 ± 0.19^b^	0.51 ± 0.14^a^
Total ash	3.04 ± 0.42^b^	73.85 ± 1.28^a^
N.F.E	5.05 ± 0.86^b^	7.65 ± 0.67^a^

Values are presented as the mean ± standard deviation of duplicate determinations.

For each row, value with the same superscript is not significantly different (*P* > 0.05) from each other.

Table 8 shows the mineral contents of the edible tissue and shell of *C*. *amnicola*. A high amount of calcium was recorded in both samples, with 47.80 ± 3.29 mg/kg in edible tissue and 124.35 ± 2.88 mg/kg in shell, and was significantly different (*P* < 0.05) from others. The shell sample had the highest concentrations of Na and CaCO_3_ at 105.38 mg/kg and 126.27 ± 2.57 mg/kg, respectively, and showed a significant difference (*P* < 0.05). K and P content appeared highest in edible tissue at *P* < 0.05.

**Table 8:** Mean concentration of mineral contents of the edible and shell part of *C*. *amnicola.*

Parameters (mg/kg)	Edible portion	Shell
Calcium	47.80 ± 3.29^b^	124.35 ± 2.88^a^
Sodium	32.26 ± 1.71^b^	105.38 ± 3.36^a^
Magnesium	2.43 ± 0.39^a^	1.73 ± 0.16^a^
Potassium	0.48 ± 0.08^a^	0.24 ± 0.03^b^
Phosphorous	0.42 ± 0.07^a^	0.12 ± 0.03^b^
Iron	8.58 ± 0.37^a^	2.57 ± 0.24^b^
CaCO_3_	63.26 ± 1.81^b^	126.27 ± 2.57^a^

Values are presented as the mean ± standard deviation of duplicate determinations.

For each row, value with the same superscript is not significantly different (*P* > 0.05) from each other.

## Discussion

Sex ratio is a crucial parameter because it aids in quantifying essential biological data of organisms needed in fisheries for sustainable management with respect to reproduction pattern, life history, regional abundance, seasonal distribution, and vulnerability of fish to fishing gear^53^. As illustrated in Table 4, the numbers of female crab samples were more abundant than male samples in all sampled months, which resulted in a favorable sex ratio for females than males and showed a significant difference (*P* < 0.05). This result obtained was in conformity with the result of Muse et al.^54^, who recorded 87 males and 108 females, yielding a sex ratio of 0.8 (males) to 1 (females) of the total 197 *C*. *amnicola* specimens collected from Akoko Beach, Lekki. Our findings were also consistent with those of Olakolu and Fakayode^55^, who recorded a higher proportion of females than males for *Callinectes amnicola*. Olapade and Sandy^56^ maintained that consistency in sex ratio enhances reproductive stability, which is required to increase the recruitment of fish and other macroinvertebrate populations. This assertion happens to contradict the significant abundance of females obtained in this study.

The length–weight relationship of aquatic macroinvertebrates through arithmetic computation of their morphometric data is important for sustainable assessment of the fisheries^41^ given that it enables accurate ascertainment of the biological conditions of aquatic species with regard to the biophysical status of their habitats. Bolger and Connolly^57^ affirmed that this fisheries management metric is also used for evaluating the wellbeing status of fish communities. Just like other fisheries indices, LWR is used to establish the fluctuations of several fish taxonomic units and other biological attributes at various life stages^41,58^^.^ LWR is particularly helpful for stock estimation because it may be used as an important paradigm for establishing fish yield or catch within a geographic area and at a given time. The major parameter of the LWR concept is the b value, which helps to quantify total body weight in relation to length^59^. In this present study, the allometric coefficient values for the 322 *Callinectes amnicola* investigated across six months were all below 3, which is an indication of a negative allometric growth pattern, as shown in Table 5. Our result is not in conformity with the findings of Lawal-Are^60^ and Atar and Secer^61^, as both researchers obtained values above 3, which denote a positive allometric for crabs. However, our finding is consistent with the result obtained by Emmanuel^62^, and Akin-Oriola et *al.* ^6^. Ricker^63^ pinpointed that the accurate value of b in relation to growth assessment should be 3; however, Froese^64^ recommended in their study that the range between 2.5 and 4.0 for the b parameter should still be considered perfect. Our monthly method of sampling and assessment might have aggravated the high dissimilarities in the b values of this present finding throughout the sampled months. Additionally, the outcome of this study may have been impacted by factors such as spawning period, food intake, and climate change, which influence environmental conditions^65^. The growth pattern may also be influenced by species age, the time and area at which the sampling is conducted, and the type of gear adopted for catch^66^. The disparities between the b values of males and females might also be due to the aforementioned elements, which have a tendency to instigate morphological variances and, as a result, uneven sizes at maturity. Frota et al.^59^ noted that varying LWR results for a specific species from a similar water body at a similar time of sampling have been reported in several studies. The discrepancies in the results of this study may be attributed to inconsistent species abundance, differences in sampling methodology, and the method adopted for evaluation. Although several morphometric characters have been adopted for the computation of LWR, in most available papers, the forkal, or total length, and total body weight have been mostly utilized for biometric-related studies^63^.

With the exception of a few months, practically all of the regression values in our study showed very weak correlations. This observation is an indication of a low positive correlation between carapace length and body weight since the correlation coefficient (r) values are very far from unity, i.e., 1. This concurs with the results obtained by Lawal-Are^60,67^ and Enin^12^ who obtained a strong positive association. The outcome of our study can be explained by the fact that a variety of factors influence the morphological development of aquatic organisms. These parameters are thus used for growth and ecological studies only in most circumstances, taking into account the time of sampling^6^.

The condition factor was calculated to estimate the well-being status of the crabs in the study area. The values obtained showed that the crabs are in good condition in their environment. There were variations or inconsistencies in the K values obtained for both sexes across all the sampled months, given that the female crabs had higher K values in some months while the male crabs had better K values in the other months, as provided in Table 5. The variation in both sexes across all the sampled months demonstrated that neither gender nor season influenced the condition factor of crabs^68^. Although age, morphology, sex and reproductive status in relation to phases of gonad development accounts for changes in K values^69,70^, The adaptation potential of animals to their environment, their intake of food, and their conversion may also have an impact on the value of condition factor^71^. Braga and Gennari-Filho^72^ asserted that a low value of k is an indication that the organism had better length than body weight growth, which might be due to inadequacies of food or spawning activity. On the other hand, a higher value of K denotes high feeding intensity and conversion efficiency, which lead to the exponential production of lipids needed for the reproduction of new species. The result of our study is in disagreement with the reports of Olakolu and Fakayode^55^ on blue swimming crabs, where the males showed a higher condition factor than the females. The abundance of female crabs bearing eggs may be the cause of the exceptionally high condition factor values for female crabs during some specific months. This is in agreement with the result of Branco and Masunari^73^, who reported a higher k and a higher weight of the female gonads of *Callinectes danae*.

The proportion of the weight of the gonads to the weight of the body is known as the gonadosomatic index (GSI). The GSI is an important biological metric used for ascertaining the precision or timing of fish spawning season, as the ovary of gravid females gets heavier and larger in size prior to spawning^74^. Our study revealed a correlation between gonad maturity, different months of the year, and the rate of ovulation with respect to the size of crabs. The monthly estimation of GSI was highest in January. Similar results were obtained for three months. The optimal breeding time is determined by crucial environmental elements like precipitation and water physico-chemistry^75^. Johal et al.^76^ reported high GSI of *Cyprinus carpio* in July, which coincides with the peak of their breeding season. This is an indication that seasons greatly impact the maturation of the ovary, which leads to subsequent changes in the gonads and body weights^77^. In the study, higher gonado-somatic index values were observed in January, March, May, and July, thereby coinciding with the occurrence of an increased percentage of mature *C*. *amnicola* during these months. This is in corroboration with the finding of Singh et al.^78^ which indicates that *C*. *amnicola* has four active spawning periods in a year.

Samya and Mohamed^79^ maintained that shellfish have long constituted a significant component of human diets, especially in coastal areas, due to their high nutritional content and accessibility at a less expensive cost. Our study evaluates the nutritional contents of both the edible tissue and shell since they are both essential to human and animal nutrition. The present study showed that the edible tissue of *C*. *amnicola* had a higher moisture content (62.57 ± 2.16%) and showed a significant difference (*P* < 0.05) from that of the shell sample (4.29 ± 0.66%). Our result was lower than the moisture contents obtained in *C*. *amnicola* by Muse et al.,^54^, and Etim,^80^. Etim^80^, who affirmed that the sampled crabs were in their spawning period, attributed the increased water contents seen in their result to the fact that organisms require more water to fill their lumen after releasing gametes during reproduction. In contrast, Davies and Jamabo^81^ maintained that organisms absorb more water to balance the osmotic condition of their cells with respect to their environments. However, high moisture content may lead to the loss of abundant polyunsaturated fatty acids while exposing the organisms to microbial deterioration. The low level of moisture content obtained in shell samples further demonstrated their resistance to quick spoilage. Nonetheless, high moisture content plays a significant role in metabolic processes and helps facilitate the emulsification of certain elements in crab tissues. Crab also contains important proteins that are rich in essential amino acids. These proteins are essential for the sustenance of life since they contain substrates needed for growth, reproduction, and synthesis in living cells^82^. The crude protein reported was high in the edible portion but not significant compared to that obtained in the shell (*P* > 0.05). Size of crabs, clean habitat, and other environmental factors, as well as availability of food, nutritional composition, and intake of food, are the major factors responsible for protein contents present in organisms. The protein content obtained in the edible portion of *C*. *amnicola* in this study was higher than the 19.82% reported by Moronkola et al.^83^ but lower than the 47% reported by Ehigiator and Akise^84^ for C. amnicola and E. radiate, respectively. Given the low-fat content of the edible tissue and shell samples used in this study, *C*. *amnicola* is classified as low-fat meat, and no significant difference (*P* > 0.05) exists between the edible and shell samples. However, the fat content in *C*. *amnicola* was lower than that reported by Kucukglumez et al.^85^ and Nalan et al.^86^. Our result is in conformity with the findings and assertions of other authors^81,85^ that shellfish belong to the low-fat category. Crab meat contains fewer calories than livestock^1^. Davies and Jamabo^81^ reported that the amount of fat retained by individual crabs within a particular period of time varies such that it is dependent on periodic change, which is instigated by environmental factors, especially temperature. Suzanne^87^ stated that fat is the main source from which energy is obtained, as it produces twice the energy generated by both carbohydrates and proteins. The availability of minerals in any organism is best deduced by the amount of total ash content present in such an organism. Ash content represents an inorganic substrate derived from any calcined organic material^88^. The findings of this study regarding the total ash content in edible tissue of *C*. *amnicola* were comparable to those of Muse et al.^54^, who also reported a low level of total ash in *C*. *amnicola* marrow. The total ash content in edible tissue of *C*. *amnicola* obtained in our study was in agreement with Woke et al.^89^, but lower than that reported by Daniel et al.^90^. The presence of a high total ash content in the shell waste of *C*. *amnicola* is an indication of mineral availability such as magnesium, calcium, potassium, and zinc^91,92^. Notably, seasonality, biological variables (maturity and reproductive stage, age, sex, body size), dietary composition and consumption, and the water quality of the aquatic habitat are the factors responsible for the amount of total ash content found in any organism.^93^. Krzynowek et al.^94^ claimed that the amount of water absorbed by any organism is influenced by the amount of crude fiber present in such an organism, but this was not the case of our finding in that the edible tissue of the crab had higher moisture than the shell, while the shell part had higher crude fiber, as seen in Table 7. Low concentrations of nitrogen-free extract were observed in this study, with the highest (7.65 ± 0.67%) in shell and the lowest (5.05 ± 0.86%) in edible tissue. This finding is consistent with the result of Bassey et al.^95^. Our result is also in line with the hypotheses of Suzanne^87^ and Eddy et al.^91^ that most sea foods, notably shellfish contains low nitrogen-free extract.

Our study revealed that Ca and its derivative compound (CaCO3), Na, Fe, and Mg are the major mineral constituents in crab edible tissues and shells (Table 8). Our outcome agrees with the finding reported by Davies and Jamabo^81^. Hughes et al.^96^pinpointed that high Ca and Na concentrations in shellfish may be caused by the makeup of the food they feed on and the kinds of minerals they take in through their gills from their environments. Aside from aiding the skeletal development and durability of shells, the presence of sodium and its derivative compounds in shells helps by regulating the pH necessary for precipitate formation^23^. The calcium content and its derivative compound (CaCO_3_) in the shell waste of this study were high and differed significantly (*P* < 0.05) from the Ca content obtained in edible tissue. However, the Ca content in the edible tissue of C. *amnicola* obtained in our study was higher compared to the earlier work obtained on C. amnicola by Davies and Jamabo^81^. Calcium is required for the proper buildup of bones and calcareous crab shells^23,97^. Moreover, calcium acts as a crucial substrate that enhances rapid activation of some specific enzymes needed for metabolic functions and hinders the occurrence of blood coagulation after damage, contraction^97^, spasticity, or stiffness of the muscles. In addition, magnesium influences the effectiveness of some enzymes and balances the complex mechanisms necessary for nerve fibers^97^. Phosphorus assists calcium in many body reactions, despite its autonomous functions. However, a relatively low amount of K, P, and Mg was obtained from the edible tissue and shell samples of *C*. *amnicola* in this study. The Mg, K, and P contents were relatively lower than those obtained in the findings of Dickson^98^. It was hypothesized that K influences the thickness of animal shells, with a reduction in shell thickness being linked with K deficiency. Phosphorus also collaborates with other vital minerals, especially Ca and Mg, in order to help build and maintain healthy bones and tissues^23,99^. The K, Mg, and P contents present in the *C*. *amnicola* samples were relatively low, although the presence of these vital minerals offers a positive correlation such that they are mutually involved in the formation processes of crabs^99–101^. The low phosphorus content obtained may be attributed to high Ca contents since both minerals are known to maintain direct and inverse relationships. The amount of any of these minerals (P and Ca) that will be present in any organism will depend on the quantity of the other. High P intake leads to loss of Ca through urination, which subsequently reduces the overall quantity of Ca present in the body and might affect the bone processes^102,103^. Aremu et al.^104^ and Nieman et al.^105^ reported that food is deemed fit and safe for consumption provided that the Ca to P ratio exceeds 1. Food with a ratio lower than 0.5 (Ca to P) is associated with health-related risk if consumed for long, while a ratio of 2 and above helps to enhance the intake of cadmium in the small intestine. The Ca/P ratio in the edible tissue of *C*. *amnicola* in our study was greater than 2, which means crab samples are rich in beneficial minerals and hence good for human consumption.

Several researchers^24,27–31^ have contributed to the trends of utilization of shell waste in several industrial and local applications towards the achievement of the target of "zero waste" in the context of the sustainable circular economy paradigm while lessening the issues of environmental pollution from the inappropriate disposal of shells. Based on the findings of this present study, it was observed that Ca, Na, and CaCO_3_ are the major mineral constituents of *C*. *amnicola,* which indicates that this crab has some impending potential and should also be categorized as secondary or supplementary materials utilized in several applications. The use of shell waste in balancing soil pH while accomplishing the goals of the circular bio-economy and reducing environmental pollution is becoming widely known due to the scientific information and practical understanding of the potential of shell waste^106,107^. This assertion is further supported by the elucidation of Summa et al.^22^ who affirmed that Barros et al.^106^ thermodynamically treated mussel shells were used and successfully used as a lime agent to balance or reconfigure soil due to the presence of alkaline in the shells, thus neutralizing the high amount of acidic elements while increasing the amount of organic substrate in the treated soil. Similarly, Fraga-Corral et al.^36^ also reported in their finding that CaCO_3_ was utilized to improve the soil’s phosphorus and oxygen content. This assertion further justifies earlier claims that the shell waste of *C*. *amnicola* examined in our study can also be integrated into several applications due to the high amount of CaCO_3_. Summa et al.^22^, in the finding of Alvarez et al.^108^ maintained that the effectiveness of processed mussel shell was comparable to that of commercial lime with respect to the fact that both liming agents equally enhanced soil pH and soil fertility level. Low cost and accessibility make mussel shells most preferred in this case. The life cycle assessment concept was adopted by Lee et al.^109^ to evaluate the overall emission from shell waste processing industries and the environmental adversity or consequences involved. It was ascertained from the outcome of their investigations that the major impact associated with waste valorization was the energy generated during calcination, pulverization, and the drying stage^22^. This indicated that the processing of shells uses less energy and, as a result, has a low impact on the environment. Summa et al.^22^ confirmed that the potential of Shell wastes has been effectively explored in the laboratory. Applications as supplements for cosmetics, green roofing, fabrics, and the biomedical industries are some of the discoveries for novel uses^22,23^. It has not been done, though, to evaluate the economic analyses, notably cost effectiveness, and the environmental-related aftermath that comes with these new discoveries. The bio-calcification formation in shellfish, particularly mollusks, is identical to the process by which bones develop or are formed in humans, indicating that shells are also crucial in osteogenesis studies. As a result, shells as a source of calcium have been distinguished and are rapidly gaining recognition in biomedical science^22,110^. Summa et al.^22^ maintained that several authors^111–113^ demonstrated the usefulness of oyster shells in the treatment of osteoporosis. Bearing in mind that the persistence of bone-related issues has been a nightmare for healthcare professionals^114^. Solutions to the bone-related problems with respect to the idea of creating biocompatible scaffolds are apparently essential.^22^. Didekhani et al.^115^ suggested that oyster shell powder should be used to rectify the low bioactivity issues related to polycaprolactone, a composite for scaffold materials. Scialla et al.^116^, Shavandi et al.^117^, Ghazali et al.^118^, and Rossi^119^ have all highlighted the significance of hydroxyapatite, which is typically a calcium phosphate mineral derived from several mollusk shells, in the remediation of bone-related problems. The potentials of hydroxyapatite (calcium phosphate) as demonstrated by the aforementioned researchers further highlighted the importance of the shell evaluated in our study since the C. amnicola shell of our finding also contains a significant amount of calcium and a reasonable quantity of phosphorus, from which the compound "phosphate" was derived.

The potential of clams, mussels, and oyster shells as sources of collagen for the possible production of cosmetics has been evaluated^118^. The valorization of mussel shell byproduct as a composite for the production of organic polymeric products has been successfully done^120^. Recently, shells from bivalve mollusks were used as substitutes for NaCl in road de-icing processes^121^, a situation that sometimes leads to water pollution if proper mitigation measures are not initiated. Morris et al.^24^ and Weiler and Scholz-Barth^122^ pinpointed in their studies that several mollusk shells have been utilized as supplementary substances for the production of roofing materials in that they prevent rapid damage to the materials due to acidic rain. The high Na content obtained in *C*. *amnicola* from our findings showed that it can also be processed and applied in such an application. Unfortunately, *C*. *amnicola* shells in the context of the circular bio-economy have yet to be optimally exploited or applied in several local and industrial applications despite their biochemical and mineral composition. The result of this study showed that the C. amnicola shell also stood a chance and could be applied in such an application due to the high level of CaCO3 obtained in its shell (Table 7).

Muir et al.^123^ claimed that calcium supplementation helps to improve the health conditions of livestock, especially the bones, and also aids in improving the quality of poultry eggshells. A comparison has been made between the quality of calcium content available in oyster shell and limestone^22^. It was therefore established after the feeding trial that the calcium found in oyster shells improves the structural and developmental formation of the bone and enhances egg production in poultry^124–127^. In the same vein, Hou et al.^128^ found that oyster shell performed better than wood ash and limestone as a replacement for calcium in terms of the growth evaluation of the experimented hens. It is worthy to note that the valorization of shell waste and the ascertainment of its effectiveness can only be quantified and achieved after successfully detaching them completely from the flesh, so as not to give credit to an animal byproduct as mentioned in EU Regulation (EC)^24^. Moreover, the authority of each member in their individual states is in charge of enforcing the "free-from-flesh" policies^129^. Thus, the valorization of shell waste in the context of sustainable all-round utilization with respect to the environment and economic evaluation must be taken into account.

From an ecotoxicological point of view, toxic metals present in contaminated soil have been subdued or remedied through the use of shell waste, as reported by Ahmad^130^, Matthew^131^, and Ok et al.^132^. In the same vein, Zhong et al.^133^ asserted that oyster shells can be used as an absorbing and remediating agent for curbing the mobility effect of toxic metals in concerned habitats. Chen et al.^134^ and Bi et al.^135^ further demonstrated that oyster shells have been utilized to alleviate the significant amount of toxic metal, with specificity to arsenic, in contaminated soils and vegetables^135^. The mixture of combusted powdered oyster shells and crushed waste product derived from cow bones has been reported by Moon et al.^136^, Zheng et al.^137^, and Hannah et al.^138^ to be an effective suppressant for the high concentration of several toxic metals in contaminated soils. These authors attributed the neutralizing or suppressing ability of these biomaterials to the presence of transiting elements, particularly CaCO3, in high concentration in shells. Fernández-Calvio et al.^139^, Garrido-Rodriguez et al.^140^, Ramrez-Pérez et al.^141^, and Ahmad et al.^142^ all highlighted the significance of mussel shells for boosting soil pH, neutralizing heavy metals, and enhancing nutrient uptake in plants. Globally, other conventional adsorption measures have also been adopted to curb the significant amount of toxic metal present in soil. However, some of the reported methods are complex and expensive to access for large-scale remediation in that they come with tasking procedures and require proper monitoring, critical scientific understanding, and practical knowledge. Additionally, Liu et al.^143^affirmed that mollusk shells have been tested as an adsorbing material for wastewater in copper-mining industries. Summa et al.^22^ mentioned that the efficiency of nanoscale mollusk shell powder as an absorbing agent has been demonstrated by Du et al.^144^.

The unending potentials of mollusk shells cannot be totally quantified, such that Gao et al.^145^, Yousefi et al.^146^, and Yen et al.^147^ claimed that oyster shell powder has been successfully used as an adsorbent for removing large concentrations of cadmium and cobalt compounds from contaminated waters. The mercury retention capacity of untreated and combusted mussel shells has been demonstrated^148^. In recent times, Summa et al.^22^ maintained that the separation techniques for the removal of several dying agents from wastewater have been established by Hussain et al.^149^, Shariffuddin et al.^150^, Elwakeel et al.^151^ and Thakul et al.^152^ through the combination of mollusk shells and glass waste products. This combination has also been tested by Henrique et al.^153^to eradicate contaminants from domestic and industrial wastewater. Summa et al.^22^ pinpointed that Quintela et al.^154^experimented with shell wastes to eliminate the remnants of antibiotics used for treating tuberculosis and also those used to inhibit the activity of living organisms in water. It has been highlighted in several studies that a few, but essential, steps need to be followed to ensure the successful application of shell waste. Some of the processes include washing, detaching from the flesh of mollusks, grinding to an appropriate size, and, lastly, the calcination process^22^. Many findings^26,155–174^ have shown that bivalve shells, in a 5–90% proportion, could be integrated in the production of several construction materials. Nevertheless, Yang et al.^175^ clearly pinpointed that the inclusion of bivalve shells as substrates in the production of building materials reduced their efficiency characteristics by over 20%, which justifies the reason why these shells are yet to be established and recommended as supplementary materials for building by large-scale manufacturing industries. Kawashima et al.^176^ maintained that, if mollusk shells undergo calcinations at an appropriate degree of temperature, the CaCO3 will be chemically converted into CaO, which can be utilized as a heterogeneous catalyst during biodiesel production and contains some essential long-chain fatty acids obtained mostly from vegetable oils and animal fats^22,177^. Mussel shells in different proportions have been used as catalysts for oil transesterification, as demonstrated by^178–183^.

## Conclusion

This finding provides comprehensive biological information, and ecological condition of blue swimming crab (*C*. *amnicola*) required for sustainability with respect to fisheries management and also highlights the importance of the studied species in the context of sustainable circular economy. The length–weight relationship of all the crabs examined showed negative allometric growth (b < 3). The lower b value obtained may be attributed to pollution status of the environment. The female crabs were more abundance which suggests that the river is a suitable ground breeding purpose. Higher "k" values in female *C*. *amnicola* than male *C*. *amnicola* might be due to lack of reproductive activity. The edible tissue of *C*. *amnicola* from our finding contains higher proteins and other essential that are essential in human and animal nutrition compare to that found in the shell. In contrary, our finding showed that the shell exoskeleton contains more essential minerals (Ca, CaCO3, Na, and Mg) known as transitional minerals compare to that found in edible parts and can be used as catalysts in several applications such as construction and roofing materials, coagulants, fillers, pigments, adsorbents, therapeutic, livestock feed, Biomedical industries, liming and fertilization and so on. Hence proper valorization of this shell waste should be encouraged rather than discarding it. Such that the ‘zero waste’ target of the sustainable circular bio-economy paradigm will further be achieved while also suppressing the environmental pollution caused by inappropriate and indiscrimination deposition of these shells in our environment.

## Data Availability

The datasets generated and/or analyzed during the study are available from the corresponding author upon reasonable request.
